# Response to “Specimen collection is essential for modern science”

**DOI:** 10.1371/journal.pbio.3002390

**Published:** 2023-11-22

**Authors:** Allison Q. Byrne

**Affiliations:** 1 Department of Environmental Science, Policy & Management, University of California, Berkeley, California, United States of America; 2 Museum of Vertebrate Zoology, University of California, Berkeley, California, Unites States of America

## Abstract

Natural history museums are vital repositories of specimens, samples and data that inform about the natural world; this Reply responds to a Formal Comment that queried whether it will ever be possible to completely do away with whole animal specimen collection, inviting open conversations about the ethical implications of specimen collection.

In my original perspective, I introduced the term “compassionate collection” to describe a practice that would involve a pivot towards nonlethal collections for natural history museums [1]. I specifically use the term compassion—to suffer with—to describe a relationship between scientists and the animals they study that recognizes animal lives as deeply meaningful; that the unique expression of lizard, or beetle, or bird that we encounter has significant value. It is my belief that if we open our hearts to this compassionate channel, we could be motivated to think creatively about how to preserve animal lives without abandoning our own curiosity about their world (which is often fueled by the same sense of wonder). This invitation has been met with resistance [2].

I recognize that my perspective and call for compassionate collection is born from my own personal journey of finding a way of doing good science without taking animal lives. For me, this has looked like developing new methods to optimize non-invasive DNA samples [3] and avoiding animal experimentation. While I agree that many past scientific discoveries made by virtue of lethal collections are important, I also think we have a responsibility to reflect on the ethical frameworks we are using when we decide how we conduct our science. My perspective was an invitation to have conversations about the hard ethical choices we must make when we decide whether to sacrifice animal lives. Therefore, when Nachman and colleagues chose to avoid the ethical dimensions of this issue in their response [2], I believe they missed the heart of the matter.

Nachman and colleagues reach the conclusion that nonlethal collections will unquestionably limit future scientific possibilities, a statement with which I disagree. For example, included in Nachman and colleagues’ list are discoveries made using collections-based wildlife surveys [4] and skin swabs from collections [5], both of which have potential nonlethal alternatives going forward that could provide even *better* data about animals and ecosystems than lethal sampling strategies. Wildlife surveys can be conducted through nonlethal means using a variety of techniques (e.g., visual encounter surveys, camera traps, audio recorders, environmental DNA sampling) that provide more robust occupancy data and have the potential for revealing behavioral and community level insights while leaving animal populations intact [6,7]. Skin swabs taken from live animals provide for more accurate pathogen detection than those taken after formalin-fixation [8]. As stated in my original piece, part of the compassionate collection approach would involve shifting priorities to build capacity to better accommodate these types of samples and the costs associated with collecting and storing them [1].

I believe that we can expand, imagine, and create new futures for natural history museums, and a natural starting point for building that future is reflecting on the relationship and responsibility we have to the animals we study. I expect that we will all land on various points along a spectrum with regards to how we weigh certain scientific outcomes versus animal lives, and I look forward to continuing the conversation and getting to know the diversity of viewpoints that exist within our community. Therefore, I would like to again offer an invitation. I invite all museum scientists to approach your science with open eyes and hearts, recognizing that your values as an individual matter in this field, just as the individual creatures you are studying matter. I encourage you to discuss how your own set of values inform and influence your research methodologies and questions. I invite us all to critically evaluate our commitment to the status quo and where that commitment comes from. These discussions can help us avoid the harms caused when moments of awe and connection in the field become moments of disconnection and heartbreak. Open dialogue about the ethics of collection will only serve to grow the important role that museums have and will continue to play in our study of the natural world.

Foundational changes in the ways we act almost always start with a change of heart. I believe that if we do center compassion in our collection and scientific practices, we will create a more inclusive, harmonious scientific community and the questions we will be able to answer will multiply, not contract. Our science will grow as we do, as the animals that live on do, and as our society must to meet the challenges we face.
